# IMGT^®^ and 30 Years of Immunoinformatics Insight in Antibody V and C Domain Structure and Function

**DOI:** 10.3390/antib8020029

**Published:** 2019-04-11

**Authors:** Marie-Paule Lefranc, Gérard Lefranc

**Affiliations:** IMGT®, the international ImMunoGeneTics information system®, University of Montpellier, CNRS, Laboratoire d’ImmunoGénétique Moléculaire LIGM, Institut de Génétique Humaine IGH, UMR 9002 CNRS-UM, 141 rue de la Cardonille, 34396 Montpellier CEDEX 5, France; glefranc@univ-montp2.fr

**Keywords:** IMGT, immunoinformatics, immunogenetics, IMGT-ONTOLOGY, IMGT Collier de Perles, IMGT unique numbering, immunoglobulin, antibody, paratope, complementarity determining region

## Abstract

At the 10th Human Genome Mapping (HGM10) Workshop, in New Haven, for the first time, immunoglobulin (IG) or antibody and T cell receptor (TR) variable (V), diversity (D), joining (J), and constant (C) genes were officially recognized as ‘genes’, as were the conventional genes. Under these HGM auspices, IMGT^®^, the international ImMunoGeneTics information system^®^, was created in June 1989 at Montpellier (University of Montpellier and CNRS). The creation of IMGT^®^ marked the birth of immunoinformatics, a new science, at the interface between immunogenetics and bioinformatics. The accuracy and the consistency between genes and alleles, sequences, and three-dimensional (3D) structures are based on the IMGT Scientific chart rules generated from the IMGT-ONTOLOGY axioms and concepts: IMGT standardized keywords (IDENTIFICATION), IMGT gene and allele nomenclature (CLASSIFICATION), IMGT standardized labels (DESCRIPTION), IMGT unique numbering and IMGT Collier de Perles (NUMEROTATION). These concepts provide IMGT^®^ immunoinformatics insights for antibody V and C domain structure and function, used for the standardized description in IMGT^®^ web resources, databases and tools, immune repertoires analysis, single cell and/or high-throughput sequencing (HTS, NGS), antibody humanization, and antibody engineering in relation with effector properties.

## 1. Introduction

IMGT^®^, the international ImMunoGeneTics information system^®^ (http://www.imgt.org), was created in June 1989 at Montpellier, by Marie-Paule Lefranc (University of Montpellier and CNRS) to characterize the genes and alleles of the antigen receptors, immunoglobulins (IG) or antibodies [1] and T cell receptors (TR) [2] and to manage the huge and complex diversity of the adaptive immune responses of the jawed vertebrates (or *gnathostomata*) from fishes to humans [3]. The creation of IMGT^®^ marked the birth of immunoinformatics, a new science at the interface between immunogenetics and bioinformatics [3]. The variable (V), diversity (D), joining (J), and constant (C) genes of the antigen receptors were officially recognized as ‘genes’, as were the conventional genes, at the 10th Human Genome Mapping (HGM10) Workshop, in New Haven, allowing IG and TR gene and allele classification. The IMGT^®^ databases and tools, built on the IMGT-ONTOLOGY axioms and concepts, bridge the gap between genes, sequences and three-dimensional (3D) structures [3]. The data accuracy and consistency are based on the IMGT Scientific chart rules generated from the axioms and concepts: IMGT^®^ standardized keywords (IDENTIFICATION axiom, concepts of identification), IMGT^®^ gene and allele nomenclature (CLASSIFICATION axiom, concepts of classification), IMGT^®^ standardized labels (DESCRIPTION axiom, concepts of description), IMGT unique numbering and IMGT Collier de Perles (NUMEROTATION axiom, concepts of numerotation) [3].

The antigen receptor IG and TR variable domains form a huge repertoire of 2.10^12^ different specificities per individual. Owing to the particularities of their synthesis that involve DNA rearrangements, there was a need for a systematic and coherent numbering of the amino acids and codons, whatever the molecule, configuration or chain type. The IMGT unique numbering was therefore a breakthrough in immunogenetics and immunoinformatics when it was defined for the first time in 1997 for the variable (V) domain [4,5,6]. The IMGT unique numbering bridges the gap between amino acid and codon sequences of any V and C and their two-dimensional (2D) and three-dimensional (3D) structures and has been fundamental in the creation of the IMGT Collier de Perles graphical representation [4,5,6]. Both concepts have allowed the standardization of the description of mutations, amino acid changes, polymorphisms, and contact analysis in the IMGT^®^ databases, tools and Web resources (http://www.imgt.org) [3].

The IMGT unique numbering was created by taking into account the high conservation of the structure of the V domain and by integrating the knowledge acquired by the analysis of multiple sources: alignment of more than 5000 sequences, literature data on the framework (FR) and complementarity determining regions (CDR), structural data from X-ray diffraction studies and characterization of the CDR hypervariable loops [4,5,6]. The standardized delimitation of the FR-IMGT and CDR-IMGT was defined based on the longest CDR1-IMGT and CDR2-IMGT found in the IMGT^®^ multiple alignments of the germline IG and TR genes and, for the rearranged CDR3-IMGT, on statistical analysis of the IG and TR rearrangements [4,5,6]. The IMGT unique numbering, originally defined for the numerotation of the IG and TR V-DOMAIN [4], was rapidly extended to the V-LIKE-DOMAIN of the immunoglobulin superfamily (IgSF) other than IG and TR [5,6], then to the constant (C) domain (C-DOMAIN of IG and TR and C-LIKE-DOMAIN of IgSF other than IG and TR) [7]. Based on the same concepts, and despite a very different structure of the groove (G) domain, the IMGT unique numbering for G domain was successfully set up for the G-DOMAIN of major histocompatibility (MH) proteins and G-LIKE-DOMAIN of MhSF other than MH [8]. 

## 2. IMGT Unique Numbering for V Domain

### 2.1. V Domain Strands and Loops

The V domain strands and loops and their IMGT^®^ positions and lengths, based on the IMGT unique numbering for V domain (V-DOMAIN of IG and TR and V-LIKE-DOMAIN) [6], are shown in Table 1. 

**Figure 1:**
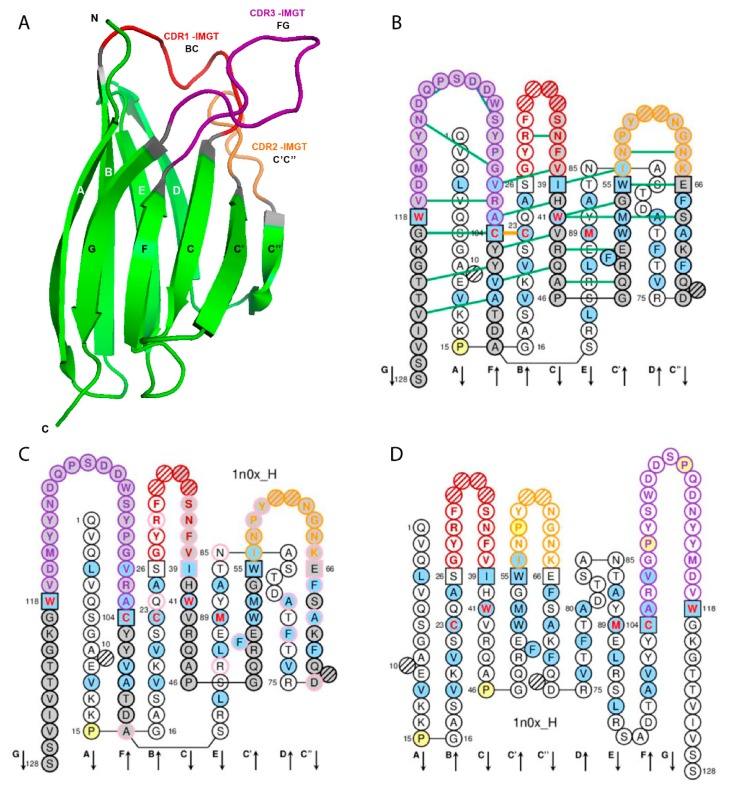
Variable (V) domain. An immunoglobulin (IG) variable heavy VH (V-DOMAIN) is shown as example. Reproduced with permission from IMGT^®^, the international ImMunoGeneTics information system^®^, http://www.imgt.org. (**A**) 3D structure ribbon representation with the IMGT strand and loop delimitations [6]. (**B**) IMGT Collier de Perles on two layers with hydrogen bonds. The IMGT Collier de Perles on two layers show, in the forefront, the GFCC′C′′ strands (forming the sheet located at the interface VH/VL of the IG) and, in the back, the ABED strands. The IMGT Collier de Perles with hydrogen bonds (green lines online, only shown here for the GFCC’C” sheet) is generated by the IMGT/Collier-de-Perles tool integrated in IMGT/3Dstructure-DB, from experimental 3D structure data [9,10,11]. (**C**) IMGT Collier de Perles on two layers generated from IMGT/DomainGapAlign [10,12,13]. Pink circles (online) indicate amino acid changes compared to the closest genes and alleles from the IMGT reference directory. (**D**) IMGT Collier de Perles on one layer. Amino acids are shown in the one-letter abbreviation. All proline (P) are shown online in yellow. IMGT anchors are in square. Hatched circles are IMGT gaps according to the IMGT unique numbering for V domain [6,14]. Positions with bold (online red) letters indicate the four conserved positions that are common to a V domain and to a C domain: 23 (1st-CYS), 41 (CONSERVED-TRP), 89 (hydrophobic), 104 (2nd-CYS) [4,5,6,7,14], and the fifth conserved position, 118 (J-TRP or J-PHE) which is specific to a V-DOMAIN and belongs to the motif F/W-G-X-G that characterizes the J-REGION [6,14] (Table 2). The hydrophobic amino acids (hydropathy index with positive value: I, V, L, F, C, M, A) and tryptophan (W) [15] found at a given position in more than 50% of sequences are shown (online with a blue background color). Arrows indicate the direction of the beta strands and their designations in 3D structures. IMGT color menu for the CDR-IMGT of a V-DOMAIN indicates the type of rearrangement, V-D-J (for a VH here, red, orange and purple) or V-J (for V-KAPPA or V-LAMBDA (not shown), blue, green and greenblue) [1]. The identifier of the chain to which the VH domain belongs is 1n0x_H (from the *Homo sapiens* b12 Fab) in IMGT/3Dstructure-DB (http://www.imgt.org). The CDR-IMGT lengths of this VH are [8.8.20] and the FR-IMGT are [25.17.38.11]. The 3D ribbon representation was obtained using PyMOL (http://www.pymol.org) and ‘IMGT numbering comparison’ of 1n0x_H (VH) from IMGT/3Dstructure-DB (http://www.imgt.org).

The V domain (V-DOMAIN and V-LIKE-DOMAIN) is composed of the A-STRAND of fifteen (or fourteen if gap at position 10) amino acids (positions 1 to 15), the B-STRAND of eleven amino acids (positions 16 to 26) with the first conserved cysteine (1st-CYS) at position 23, the BC-LOOP (positions 27 to 38; the longest BC loops have 12 amino acids), the C-STRAND of eight amino acids (positions 39 to 46) with the tryptophan (CONSERVED-TRP) at position 41, the C’-STRAND of nine amino acids (positions 47 to 55), the C’C”-LOOP (positions 56 to 65; the longest C’C” loops have 10 amino acids), the C”-STRAND of nine (or eight if gap at position 73) amino acids (positions 66 to 74), the D-STRAND of ten (or eight if gaps at positions 81 and 82) amino acids (positions 75 to 84), the E-STRAND of twelve amino acids (positions 85 to 96) with a conserved hydrophobic amino acid at position 89, the F-STRAND of eight amino acids (positions 97 to 104) with the second conserved cystein (2nd-CYS) at position 104, the FG-LOOP (positions 105 to 117; these positions correspond to a FG loop of 13 amino acids) and the G-STRAND of eleven (or ten) amino acids (positions 118 to 128) (Table 1, Figure 1). In the IG and TR V-DOMAIN, the G-STRAND is the C-terminal part of the J-REGION, with J-PHE or J-TRP 118 and the canonical motif F/W-G-X-G (J-MOTIF) at positions 118–121 [6] (Table 1). 

In the IG and TR V-DOMAIN, the structurally conserved antiparallel beta strands are also designated as framework regions (FR-IMGT) whereas the loops are designated as complementarity determining regions (CDR-IMGT) [6]. Strands A and B correspond to the FR1-IMGT (positions 1 to 26), strands C and C’ to the FR2-IMGT (positions 39 to 55), strands C”, D, E, and F to the FR3-IMGT (positions 66 to 104) and strand G to the FR4-IMGT (positions 118 to 128). The BC, C’C”, and FG loops correspond to the CDR1-IMGT, CDR2-IMGT and CDR3-IMGT, respectively [6] (Table 1, Figure 1).

IMGT anchors belong to the strands (or FR-IMGT) and represent ‘anchors’ supporting the three BC, C’C” and FG loops (or CDR-IMGT). V domain anchor positions are positions 26 and 39, 55 and 66, and 104 and 118, shown in square in IMGT Colliers de Perles. In a V-DOMAIN, the 2nd-CYS at position 104 (F strand) and J-PHE or J-TRP at position 118 (G strand) are anchors of the FG loop (or CDR3-IMGT) [5,6]. 

The loop length (number of amino acids (or codons), that is number of occupied positions) is a crucial and original concept of IMGT-ONTOLOGY [3]. The lengths of the CDR1-IMGT (BC), CDR2-IMGT (C’C”), and CDR3-IMGT (FG) characterize the V-DOMAIN. Thus, the length of the three CDR-IMGT (loops) is shown, in number of amino acids (or codons), into brackets and separated by dots. For example [8.8.20] means that the CDR1-IMGT (BC), CDR2-IMGT (C’C”), and CDR3-IMGT (FG) have lengths of 8, 8, and 20 amino acids (or codons), respectively. The JUNCTION of an IG or TR V-DOMAIN includes the anchors 104 and 118 and is therefore two amino acids longer than the corresponding CDR3-IMGT (positions 105–117) [5,6].

### 2.2. IMGT Gaps and Additional Positions

IMGT gaps are shown by dots in IMGT Protein displays [1,2,9,10] and by hatched circles or squares in IMGT Colliers de Perles for V domain and correspond to unoccupied positions according to the IMGT unique numbering for V domain [6] (Figure 1). For BC, C’C” or FG loops shorter than 12, 10, and 13 amino acids, respectively, gaps are created at the apex (positions hatched in IMGT Collier de Perles, or not shown in structural data representations). The gaps are placed at the apex of the loop with an equal number of amino acids (or codons) on both sides if the loop length is an even number, or with one more amino acid (or codon) in the left part if it is an odd number. For example, for FG loops shorter than 13 amino acids, gaps are created from the apex of the loop, in the following order: 111, 112, 110, 113, 109, etc. (IMGT^®^ http://www.imgt.org, IMGT Scientific chart > Numbering > IMGT unique numbering for V-DOMAIN and V-LIKE-DOMAIN).

For CDR3-IMGT (FG) loop longer than 13 amino acids, additional positions are created, between positions 111 and 112 at the top of the loop, in the following order: 112.1, 111.1, 112.2, 111.2, 112.3, etc. [6] (IMGT^®^ http://www.imgt.org, IMGT Scientific chart > Numbering > IMGT unique numbering for V-DOMAIN and V-LIKE-DOMAIN).

## 3. IMGT Unique Numbering for C Domain

### 3.1. C Domain Strands, Loops, and Turns

The C domain strands, turns and loops and their IMGT positions and lengths, based on the IMGT unique numbering for C domain (C-DOMAIN of IG and TR and C-LIKE-DOMAIN) [7], are shown in Table 2. 

**Figure 2:**
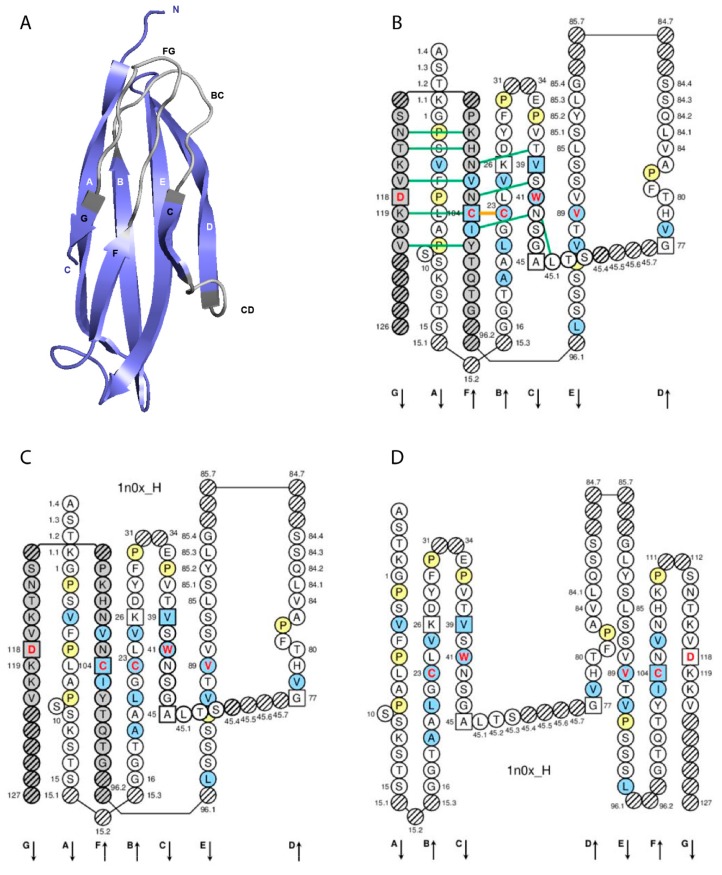
Constant (C) domain. An IG IGHG1 CH1 (C-DOMAIN) is shown as example. Reproduced with permission from IMGT^®^, the international ImMunoGeneTics information system^®^, http://www.imgt.org. (**A**) 3D structure ribbon representation with the IMGT strand and loop delimitations [7]. (**B**) IMGT Collier de Perles on two layers with hydrogen bonds. The IMGT Colliers de Perles on two layers show, in the forefront, the GFC strands and, in the back, the ABED strands (located at the interface CH1/CL of the IG), linked by the CD transversal strand. The IMGT Collier de Perles with hydrogen bonds (green lines online, only shown here for the GFC sheet) is generated by the IMGT/Collier-de-Perles tool integrated in IMGT/3Dstructure-DB, from experimental 3D structure data [9,10,11]. (**C**) IMGT Collier de Perles on two layers from IMGT/DomainGapAlign [10,12,13]. (**D**) IMGT Colliers de Perles on one layer. Amino acids are shown in the one-letter abbreviation. All proline (P) are shown online in yellow. IMGT anchors are in square. Hatched circles are IMGT gaps according to the IMGT unique numbering for C domain [7,14]. Positions with bold (online red) letters indicate the four conserved positions that are common to a V domain and to a C domain: 23 (1st-CYS), 41 (CONSERVED-TRP), 89 (hydrophobic), 104 (2nd-CYS) [4,5,6,7,14], and position 118 which is only conserved in V-DOMAIN. The identifier of the chain to which the CH1 domain belongs is 1n0x_H (from the *Homo sapiens* b12 Fab, in IMGT/3Dstructure-DB, http://www.imgt.org). The 3D ribbon representation was obtained using PyMOL and ‘IMGT numbering comparison’ of 1n0x_H (CH1) from IMGT/3Dstructure-DB (http://www.imgt.org).

The C domain (C-DOMAIN and C-LIKE-DOMAIN) is composed by the A-STRAND of fifteen (or fourteen if gap at 10) amino acids (positions 1 to 15), the AB-TURN (additional positions 15.1, 15.2, and 15.3; the longest AB-TURN have 3 amino acids), the B-STRAND of eleven amino acids (positions 16 to 26) with the 1st-CYS at position 23, the BC-LOOP (positions 27 to 31, 34 to 38), the C-STRAND of seven amino acids (positions 39 to 45) with the CONSERVED-TRP at position 41, the CD-STRAND of one to nine amino acids (additional positions 45.1 to 45.9), the D-STRAND of eight (or seven if gap at 82) amino acids (positions 77 to 84), the DE-TURN (additional positions 84.1 to 84.7 and 85.7 to 85.1, corresponding to 14 amino acids), the E-STRAND of twelve amino acids (positions 85 to 96) with a conserved hydrophobic amino acid at position 89, the EF-TURN (additional positions 96.1 and 96.2, corresponding to 2 amino acids), the F-STRAND of eight amino acids (positions 97 to 104) with the 2nd-CYS at position 104, the FG-LOOP (positions 105 to 117, these positions corresponding to a FG loop of 13 amino acids), and the G-STRAND of eleven (or less) amino acids (positions 118 to 128) [7] (Table 2, Figure 2).

IMGT anchors belong to the strands and represent, for the C domains, anchors for the BC and FG loops and by extension to the CD strand (as C domains do not have the C’-C’’ loop) [7]. Anchor positions are shown in square in IMGT Colliers de Perles. C domain anchor positions are positions 26 and 39, 45 and 77 (anchors of the CD strand), and 104 and 118 [7].

### 3.2. C Domain and V Domain Comparison

The A-STRAND and B-STRAND of the C domain are similar to those of the V domain [7]. The longest BC-LOOP of the C domain have 10 amino acids (missing positions 32 and 33), instead of 12 amino acids in the V domain. The C-STRAND and the D-STRAND of the C domain are shorter of one position (46) and two positions (75, 76), respectively, compared to those of the V domain. The transversal CD-STRAND is a characteristic of the C domain (a V domain has instead two antiparallel beta strands C’-STRAND and C”-STRAND linked by the C’C”-LOOP). The E-STRAND, F-STRAND and G-STRAND of the C domain are similar to those of the V domain [7] (IMGT^®^ http://www.imgt.org, IMGT Scientific chart > Numbering > IMGT unique numbering for C-DOMAIN and C-LIKE-DOMAIN).

### 3.3. IMGT Gaps and Additional Positions

IMGT gaps are shown by dots in IMGT Protein displays and by hatched circles or squares in IMGT Colliers de Perles for C domain and correspond to unoccupied positions according to the IMGT unique numbering for C domain [7]. 

The longest BC-LOOP of the C domain have 10 amino acids (missing positions 32 and 33, that are a feature of the C domain are not shown in the IMGT Colliers de Perles and IMGT Protein displays for C domain). For BC loops shorter than 10 amino acids, gaps are created from the apex in the following order 34, 31, 35, 30, 36, etc. The FG-LOOP of the C domain is similar to that of the V domain. Gaps for FG loops shorter than 13 amino acids and additional positions for FG loops longer than 13 amino acids, are created following the same rules as those of the V domain.

Additional positions in the C domain define the AB-TURN, DE-TURN and EF-TURN (Table 2). For AB-TURN shorter than 3 amino acids, gaps are created (hatched in IMGT Colliers de Perles, or not shown in structural data representations) in a decreasing ordinal manner. For DE-TURN shorter than 14 amino acids, gaps are created in the following order: 85.7, 84.7, 85.6, 84.6, 85.5, etc. For EF-TURN shorter than 2 amino acids, gaps are created in the following order: 96.2, 96.1 [7].

## 4. IMGT^®^ V and C Domain Insight for Antibody Humanization and Engineering

### 4.1. Antibody Humanization

#### 4.1.1. CDR-IMGT Delimitation for Grafting

The objective of antibody humanization is to graft at the DNA level the CDR of an antibody V domain, from mouse (or other species) and of a given specificity, onto a human V domain framework, thus preserving the specificity of the original (murine or other species) antibody while decreasing its immunogenicity [16]. IMGT/DomainGapAlign [10,12,13] is the reference tool for antibody humanization design based on CDR grafting: (i) it precisely defines the CDR1-IMGT, CDR2-IMGT and CDR3-IMGT to be grafted, and (ii) it helps selecting the most appropriate human FR-IMGT by providing the alignment of the mouse (or other species) V-DOMAIN amino acid sequence with the closest germline *Homo sapiens* V-REGION and J-REGION. 

Analyses performed on humanized therapeutic antibodies underline the importance of a correct delimitation of the CDR and FR. As an example, two amino acid changes were required in the first version of the humanized VH of alemtuzumab, in order to restore the specificity and affinity of the original rat antibody. The positions of these amino acid changes (S28>F and S35>F) are now known to be located in the CDR1-IMGT and should have been directly grafted, but at the time of this mAb humanization they were considered as belonging to the FR according to the Kabat numbering [17]. In contrast, positions 66–74 were, at the same time, considered as belonging to the CDR according to the Kabat numbering, whereas they clearly belong to the FR2-IMGT and the corresponding sequence should have been ‘human’ instead of being grafted from the ‘rat’ sequence. 

#### 4.1.2. Amino Acid Interactions between FR-IMGT and CDR-IMGT

IMGT Colliers de Perles from crystallized 3D structures in IMGT/3Dstructure-DB [9,10,11] highlight two conserved hydrogen bonds between FR-IMGT and CDR-IMGT positions: the first one between FR2-IMGT 39 and CDR2-IMGT 56 (or 57), and the second one between FR2-IMGT 40 and CDR3-IMGT 105 (Figure 1B). Antibody engineering and humanization should therefore preserve these bondings which stabilize the loops. It is also worthwhile to note that, in VH CDR3, the stem of the CDR3 loop is stabilized by a conserved salt bridge between R106 (arginine contributed by the 3’V-REGION) and D116 (aspartate contributed by the 5’J-REGION of the *Homo sapiens* IGHJ2, IGHJ3, IGHJ4, IGHJ5 or IGHJ6 (IMGT^®^ http://www.imgt.org, IMGT Repertoire > Alignments of alleles > IGHJ > human (*Homo sapiens*) Overview).

#### 4.1.3. V-DOMAIN Contact Analysis and Paratope

**Figure 3:**
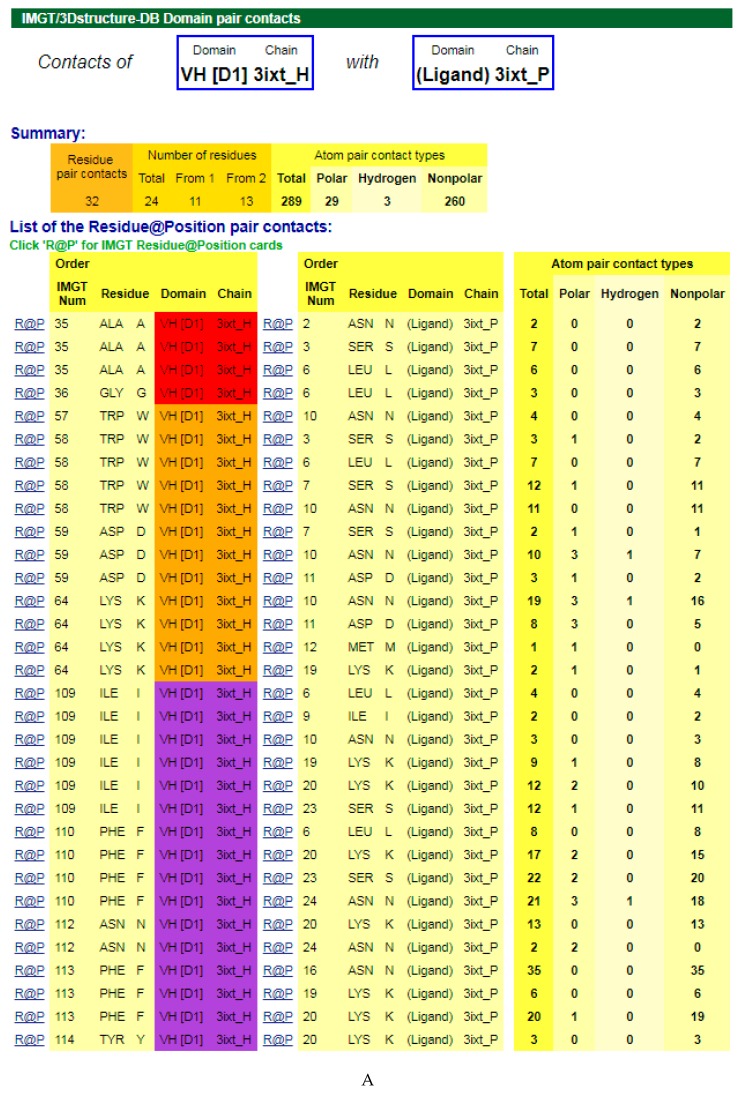

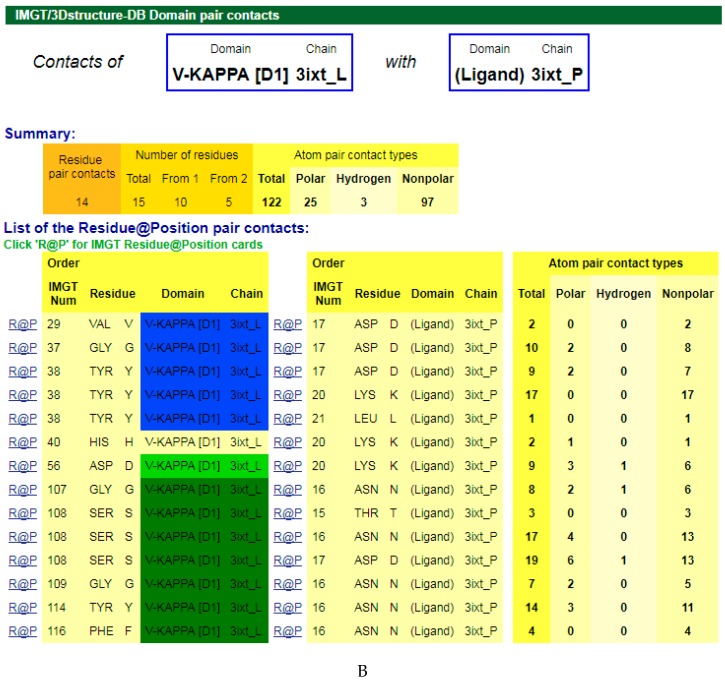
V-DOMAIN Contact analysis results. Reproduced with permission from IMGT^®^, the international ImMunoGeneTics information system^®^, http://www.imgt.org. (**A**) IMGT/3Dstructure-DB domain pair contacts between the VH domain of motavizumab (3ixt_H) and the Fusion glycoprotein F1 (ligand) (3ixt_P). (**B**) IMGT/3Dstructure-DB Domain pair contacts between the V-KAPPA domain of motavizumab (3ixt_L) and the Fusion glycoprotein F1 (ligand) (3ixt_P). ‘Polar’, ‘Hydrogen bonds’, and ‘Nonpolar’ were selected prior to display, in ‘Atom contact types’. Amino acids belonging to the CDR1-IMGT, CDR2-IMGT and CDR3-IMGT are colored according to the IMGT color menu (red, orange, and purple, respectively, for VH; blue, light green and green, respectively, for V-KAPPA). In this 3D structure, all but one of the amino acids contacting the antigen belong to the CDR-IMGT. Clicking on R@P gives access to the IMGT Residue@Position cards [9,10,11].

The amino acids of the V-DOMAIN CDR-IMGT involved in the contacts with the antigen can be visualized in IMGT/3Dstructure-DB Contact analysis [9,10,11] which provides extensive information on the atom pair contacts. Domain pair contacts (‘DomPair’) provide information on the contacts between a pair of partners (for examples, between the VH domain of motavizumab (3ixt_H chain) and the ligand (3ixt_P chain), or between the V-KAPPA domain of motavizumab (3ixt_L chain) and the ligand (3ixt_P chain) (Figure 3) [9,10,11]. Clicking on R@P gives access to the IMGT Residue@Position cards [9,10,11]. 

The IG paratope of 3ixt (motavizumab Fab) comprises AA of the VH (3ixt_H chain) and of the V-KAPPA (3ixt_L chain). Fifteen AA of the IG, eight from VH and seven from V-KAPPA, form the paratope (IMGT^®^ http://www.imgt.org, IMGT/3Dstructure-DB > Query 3ixt > Paratope and epitope). The IMGT Colliers de Perles show that eight (out of the eight VH positions of the paratope) belong to the VH CDR-IMGT: A35 (CDR1-IMGT); W58, D59, and K64 (CDR2-IMGT); I109, F110, N112 and F113 (CDR3-IMGT), and that similarly seven (out of the seven V-KAPPA positions of the paratope) belong to the V-KAPPA CDR-IMGT: G37 and Y38 (CDR1-IMGT); D56 (CDR2-IMGT); G107, S108, G109, and Y114 (CDR3-IMGT) [9,10,11].

#### 4.1.4. Potential Immunogenicity and Physicochemical Properties

The number of amino acid differences in the FR-IMGT and CDR-IMGT is one of the criteria to evaluate the potential immunogenicity. The framework of a VH domain comprises 91 positions (25, 17, 38, and 11 positions for FR1-, FR2-, FR3-, and FR4-IMGT, respectively), whereas the framework of a VL domain comprises 89 positions (26, 17, 36, 10 positions for FR1-, FR2-, FR3-, and FR4-IMGT, respectively) [9,10,11]. The amino acid (AA) changes are described for the hydropathy (three classes), volume (five classes) and physicochemical properties (11 classes) [15] (IMGT^®^ http://www.imgt.org, IMGT Aide-mémoire > IMGT classes of the 20 common amino acids). S40 > G (+ + -) means that the two AA involved in the change (S > G) at codon 40 belong to the same hydropathy (+) and volume (+) classes but to different physicochemical properties (-) classes [15]. This qualification of AA replacement has led to the identification of four types of AA changes: very similar (+ + + ), similar (+ + -, + - +), dissimilar (- - +, - + -, + - -), and very dissimilar (- - -).

#### 4.1.5. V-DOMAIN CDR-IMGT Lengths and Canonical Structures

For V-DOMAIN comparison including sequences and structures, the CDR1-IMGT and CDR2-IMGT lengths are more informative than the "canonical structures" (IMGT^®^ http://www.imgt.org, IMGT Repertoire (IG and TR) > 2D and 3D structures > CDR1-IMGT (summary) and correspondence with "canonical structures": human *(Homo sapiens)* and mouse *(Mus musculus)* Immunoglobulins; ibid CDR2-IMGT). Indeed, (1) most identified (15 out of 19) canonical structures correspond to a given CDR-IMGT length, (2) only two CDR-IMGT lengths have two canonical structures (CDR1-IMGT of nine AA of IGLV, and CDR2-IMGT of eight AA of IGHV), (3) canonical structures have not been identified for every CDR-IMGT length, (4) many ‘variants’ are described in the literature, based only on sequences and without experimental evidence, (5) canonical structures cannot be identified for CDR3 owing to their diversity in lengths and sequences and to their flexibility, and (6) canonical structure identification is reliable only if 3D structures are known [14]. Thus, the CDR-IMGT length is the most accurate way to define the three CDR, while working on sequences, that information being completed with characteristics Residue@Position, if necessary [9,10,11]. 

### 4.2. IGHG1 Alleles and G1m Allotypes

Allotypes are polymorphic markers of an IG subclass that correspond to amino acid changes and are detected serologically by antibody reagents [18]. In therapeutic antibodies (human, humanized, or chimeric), allotypes may represent potential immunogenic residues [19], as demonstrated by the presence of antibodies in individuals immunized against these allotypes [18]. The allotypes of the human heavy gamma chains of the IgG are designated as Gm (for gamma marker). The allotypes G1m, G2m, and G3m are carried by the constant region of the gamma1, gamma2 and gamma3 chains, encoded by the IGHG1, IGHG2 and IGHG3 genes, respectively [18]. The gamma1 chains may express four G1m alleles (combinations of G1m allotypes): G1m3, G1m3,1, G1m17,1, and G1m17,1,2 (and in Negroid populations three additional G1m alleles, G1m17,1,27, G1m17,1,28, and G1m17,1,27,28) [18] (Table 3). The C region of the G1m3,1, G1m17,1, and G1m17,1,2 chains differ from that of the G1m3 chains by two, three and four amino acids, respectively [18]. The correspondence between the G1m alleles and IGHG1 alleles is shown in Table 3. Thus, IGHG1*01, IGHG1*02 and IGHG1*05 are G1m17,1, IGHG1*03 is G1m3, IGHG1*04 is G1m17,1,27 and IGHG1*08p is G1m3,1. In the IGHG1 CH1, the lysine at position 120 (K120) in strand G corresponds to the G1m17 allotype [18] (Figure 2D). The isoleucine I103 (strand F) is specific of the gamma1 chain isotype. If an arginine is expressed at position 120 (R120), the simultaneous presence of R120 and I103 corresponds to the expression of the G1m3 allotype [18]. For the gamma3 and gamma4 isotypes (which also have R120 but T in 103), R120 only corresponds to the expression of the nG1m17 isoallotype (an isoallotype or nGm is detected by antibody reagents that identify this marker as an allotype in one IgG subclass and as an isotype for other subclasses) [18]. In the IGHG1 CH3, the aspartate D12 and leucine L14 (strand A) correspond to G1m1, whereas glutamate E12 and methionine M14 correspond to the nG1m1 isoallotype [18] (Table 3). A glycine at position 110 corresponds to G1m2, whereas an alanine does not correspond to any allotype (G1m2-negative chain) (Table 3). Therapeutic antibodies are most frequently of the IgG1 isotype, and to avoid a potential immunogenicity, the constant region of the gamma1 chains are often engineered to replace the G1m3 allotype by the less immunogenic G1m17 (CH1 R120 > K) (G1m17 is more extensively found in different populations) [18]. 

### 4.3. Only-Heavy-Chain Antibodies

#### 4.3.1. Dromedary IgG2 and IgG3 Only-Heavy-Chain Antibodies

Two IgG antibody formats are expressed in the dromedary or Arabian camel (*Camelus dromedarius*) and in Camelidae in general: the conventional IG (with two identical heavy gamma chains associated to two identical light chains) and the ‘only-heavy-chain’ IG (no light chain, and only two identical heavy gamma chains lacking CH1) [20]. The Camdro (for *Camelus dromedarius* in the 6-letter species abbreviation) IGHV3 genes belong to two sets based on four amino acid changes which are characteristic of each set [21]. The first set of IGHV3 genes is expressed in conventional tetrameric IgG1 that constitute 25% of circulating antibodies. The second set is expressed in the only-heavy-chain antibodies, IgG2 and IgG3 that constitute 75% of the circulating antibodies [20]. The four amino acid changes are located in the FR2-IMGT at positions 42, 49, 50 and 52, the first position 42 is in the C strand and the three others (49, 50 and 52) in the C’ strand (Figure 1). They belong to the (GFCC’C”) sheet at the hydrophobic VH-VL interface in conventional antibodies of Camelidae as well as of any vertebrate species whereas, in camelid only-heavy-chain antibodies (no light chains, and therefore no VL), these positions are exposed to the environment with, through evolution, a selection of hydrophilic amino acids. 

The respective heavy gamma2 and gamma3 chains are both characterized by the absence of the CH1 domain owing to a splicing defect [22]. It is the absence of CH1 which is responsible for the lack of association of the light chains. Only-heavy-chain antibodies is a feature of the Camelidae IG as they have also been found in the Bactrian camel (*Camelus bactrianus*) of Central Asia and in the llama (*Lama glama*) and alpaca (*Vicugna pacos*) of South America. The genetic event (splicing defect) responsible for the lack of CH1 occurred in their common ancestor before the radiation between the ‘camelini’ and ‘lamini’, dating approximately 11 million years (Ma) ago. 

The V domains of Camelidae only-heavy-chain antibodies have characteristics for potential pharmaceutical applications (e.g., easy production and selection of single-domain format, extended CDR3 with novel specificities and binding to protein clefts). They are designated as VH_H_ when they have to be distinguished from conventional VH (the sequence criteria is based on the four amino acids at positions 42, 49, 50 and 52). The term ‘nanobody’ initially used for describing a single-domain format antibody is not equivalent to VH_H,_ as it has been used for V domains other than VH_H_ and for constructs containing more than one V domain (VH and/or VH_H)_ (e.g., caplacizumab, ozoralizumab) (IMGT^®^ http://www.imgt.org, IMGT Repertoire > Locus and Genes > Gene tables; ibid., The IMGT Biotechnology page > Characteristics of the camelidae (camel, llama) antibody synthesis; ibid. IMGT/mAb-DB > caplacizumab; ibid. IMGT/mAb-DB > ozoralizumab).

#### 4.3.2. Human Heavy Chain Diseases (HCD)

The camelidae only-heavy-chain antibodies synthesis is remarkably reminiscent of what is observed in human heavy chain diseases (HCD). These proliferative disorders of B lymphoid cells produce truncated monoclonal immunoglobulin heavy chains which lack associated light chains. In most HCD, the absence of the heavy chain CH1 domain by deletion or splicing defect may be responsible for the lack of assembly of the light chain [23]. Similar observations have also been reported in mouse variants [23]. (IMGT^®^ http://www.imgt.org, IMGT Education > Tutorials > Molecular defects in Immunoglobulin Heavy Chain Diseases (HCDs))

#### 4.3.3. Nurse Shark IgN

A convergence mechanism in evolution is observed in nurse shark (*Ginglymostoma cirratum*, ‘Gincir’ in the 6-letter species abbreviation) IgN antibodies (previously IgNAR, ’immunoglobulin new antigen receptor’) [24] which are only-heavy-chain antibodies (homodimeric heavy nu chains without CH1, and no associated light chains). The IGHV genes expressed in the Gincir heavy nu chains belong to the IGHV2 subgroup and are characterized by the absence of the CDR2-IMGT owing to a deletion that encompasses position 54 to 67. The Gincir IGH genes are organized in duplicated cassettes, and those that express IgN comprise Gincir IGHV2 subgroup genes and an IGHN constant gene. (IMGT^®^ http://www.imgt.org, IMGT Repertoire (IG and TR) > Protein displays: nurse shark (*Ginglymostoma cirratum*) IGHV).

## 5. IGHG CH Properties and Antibody Engineering

### 5.1. N-Linked Glycosylation Site CH2 N84.4

A N-linked glycosylation site is present in the CH2 domain of the constant region of the human IG heavy chains of the four IgG isotypes. The N-linked glycosylation site belongs to the classical N-glycosylation motif N-X-S/T (where N is asparagine, X any amino acid except proline, S serine, T threonine) and is defined as CH2 N84.4. As shown in the IMGT Collier de Perles, this asparagine is localized at the DE turn. The IMGT unique numbering has the advantage of identifying the C domain (here, CH2) and, in the domain, the amino acid and its localization (here, N84.4) which can be visualized in the IMGT Collier de Perles and correlated with the 3D structure [25,26,27] (IMGT^®^ http://www.imgt.org, The IMGT Biotechnology page > Glycosylation (IMGT Lexique)).

### 5.2. Interface Ball-and-Socket-Like Joints

The 3D structure comparison, between *Homo sapiens* IGHG1 Fc and IGHG2 Fc, of the CH2 and CH3 domain interface revealed that in all IGHG Fc the movement of the CH2 results from a pivoting around a highly conserved ball-and-socket-like joint [28]. Using the IMGT numbering, the CH2 L15 side chain (last position of the A strand, next to the AB turn) (the ball) interacts with a pocket (the socket) formed by CH3 M107, H108, E109, and H115 (FG loop) [25]. These amino acids are well conserved between the gamma isotypes and the IGHG genes and alleles except for IGHG3 H115 that shows a polymorphism associated to different G3m allotypes [18]. This ball-and-socket-like joint is a structural feature similar but reversed to that previously described at the VH and CH1 domain interface [29], in which the VH L12, T125 and S127 form the socket whereas the CH1 F29 and P30 (BC loop) form the ball. 

### 5.3. Knobs-Into-Holes CH3 for the Obtaining of Bispecific Antibodies

The knobs-into-holes methodology has been proposed for obtaining bispecific antibodies [30]. The aim is to increase interactions between the CH3 domain of two gamma1 chains that belong to antibodies with a different specificity. Two amino acids, CH3 T22 (B strand) and Y86 (E strand), which belong to the [ABED] sheet, at the interface of the two *Homo sapiens* IGHG1 CH3 domains [25], were selected for amino acid changes. Interactions of these two amino acids are described in ‘Contact analysis’ in IMGT/3Dstructure-DB [9,10,11]. The knobs-into-holes methodology consists of an amino acid change on one CH3 domain (e.g., T22>Y) that creates a knob, and another amino acid change on the other CH3 domain (e.g., Y86>T) that creates a hole, thus favoring increased interactions between the CH3 of the two gamma1 chains at both positions 22 and 86 [30] (IMGT^®^ http://www.imgt.org, The IMGT Biotechnology page > Knobs-into-holes).

### 5.4. IGHG Engineered Variants and Effector Properties

Amino acids in the IGHG constant regions of the IG heavy chains are frequently engineered to modify the effector properties of the therapeutic monoclonal antibodies. Amino acids changes are engineered at positions involved in antibody-dependent cellular (ADCC), antibody-dependent cellular phagocytosis (ADCP), complement-dependent cytotoxicity (CDC), half-life increase, half-IG exchange, and B cell inhibition by coengagement of antigen and FcγR on the same cell (IMGT^®^ http://www.imgt.org, The IMGT Biotechnology page > Amino acid positions involved in ADCC, ADCP, CDC, half-life and half-IG exchange).

The IMGT engineered variant nomenclature (Table 4) has been set up for an easier comparison between engineered antibodies. The IMGT engineered variant name comprises the species, the gene name, the letter ’v’ with a number (e.g., *Homo sapiens* IGHG1v1), and then the domain(s) with AA change(s) defined by the letter of the novel AA and position in the domain (e.g., CH2, P1.4). The IMGT engineered variants are classified by comparison with the allele *01 of the gene and, if the effects are independent on the alleles, as a reference for the description of the amino acid (AA) changes for the other alleles. In those cases, the same variant (v) number is used for any allele of the same gene in the same species. 

## 6. Conclusions

IMGT^®^, created in 1989 with the official recognition of IG and TR genes, is at the origin of immunoinformatics [3]. The concepts of classification (nomenclature and IG and TR gene and allele names, CLASSIFICATION axiom) were soon followed by the concepts of identification (standardized IMGT keywords, IDENTIFICATION axiom) and the concepts of description (standardized IMGT labels, DESCRIPTION axiom) which led to the implementation of IMGT/LIGM-DB, the first IMGT sequence database demonstrated online at the 9th International Congress of Immunology (ICI), San Francisco (USA), in July 1995. It took two more years to conceive the concepts of numerotation, IMGT unique numbering and IMGT Collier de Perles (NUMEROTATION axiom) which bridge sequences and structures of V and C domain (at the amino acid and codon levels) [3]. Interestingly, the first IMGT Collier de Perles, created manually in December 2007, not only identified conflicts between the SEQRES and ATOM lines of the PDB file but also the absence of a serine at position 93, demonstrating that indeed sequence and structure were bridged using the IMGT unique numbering (http://www.imgt.org/IMGTrepertoire/2D-3Dstruct/2D-representations/mouse/IG/E5.2Fv/ighV-D-J_E5_2Fv.html).

The IMGT^®^ databases, tools and web resources have been built to manage immunogenetics knowledge and immunoinformatics, based on the IMGT Scientific chart rules generated from the IMGT-ONTOLOGY axioms and concepts [3]. Nowadays, IMGT^®^ provides standardized and integrated databases, tools and web resources for IG and TR, from gene to structure and function [31,32,33,34,35,36,37,38,39,40,41,42,43]. The same concepts and insights for the V and C domain, are used for all vertebrate species with jaws (*gnathostomata*), from fishes to humans, providing a unique resource whatever the antigen receptor, the chain type and the taxon, for study of the adaptive immune response [3]. IG repertoire analysis and therapeutic antibody development represent two major current fields of immunoinformatics, involving V and C domains, in fundamental, pharmaceutical and medical research. High throughput (HTS) data obtained by NGS has made IMGT^®^ standardization, developed originally to handle the huge diversity of the immune repertoires, more needed as ever. Since October 2010, the IMGT/HighV-QUEST web portal has been a paradigm for the characterization of the V domain diversity and expression and the identification of the IMGT clonotypes (AA) [44,45,46]. Statistical comparison of the V domain and IMGT clonotype (AA) diversity and expression between two sets can be performed using the IMGT/StatClonotype package [47,48]. NGS analysis of V domain provides immunoprofiling in normal (infectious diseases, vaccination, aging) or pathological (leukemias, lymphomas, myelomas, immunodeficiencies) conditions. An IMGT/HighV-QUEST novel functionality includes, with the same high-quality criteria, the analysis of the two V domains of single chain Fragment variable (scFv) from phage display combinatorial libraries) [49,50,51].

The therapeutic monoclonal antibody engineering field represents the most promising potential in medicine. Standardized genomic and expressed sequence, structure and interaction analysis of IG is crucial for a better molecular understanding and comparison of the mAb specificity, affinity, half-life, Fc effector properties, and potential immunogenicity. IMGT/3Dstructure-DB provides a standardized description and antibody structure/contact analysis characterization, at the V and C domain level, at the chain level (with the ‘chimeric’ and ‘humanized’ added as ‘taxon’), and at the receptor level. Amino acids (or codons) changes (either polymorphic or resulting from engineering are identified. The structural unit is the V or C domain, with for regions (hinge, linker, CHS). This modular characterization per domain (and/or region) provides a great flexibility and is applicable to any novel format of antibody engineering [52,53,54,55,56,57,58,59]. IMGT concepts have been integrated in the Encyclopedia of Systems Biology [60,61,62,63]. The CDR-IMGT lengths are now required for mAb INN applications and are included in the World Health Organization International Nonproprietary Name WHO INN definitions [64], bringing a new level of standardized information in the comparative analysis of therapeutic antibodies.

## Figures and Tables

**Table 1:** V domain strands and loops, IMGT (IMGT^®^, the international ImMunoGeneTics information system^®^) positions and lengths, based on the IMGT unique numbering for V domain (V-DOMAIN and V-LIKE-DOMAIN) [6]. FR: framework; CDR: complementarity determining regions.

V Domain Strands and Loops ^a^	IMGT Positions	Lengths ^b^	Characteristic Residue@Position ^c^	V-DOMAIN FR-IMGT and CDR-IMGT
A-STRAND	1–15	15 (14 if gap at 10)		FR1-IMGT
B-STRAND	16–26	11	1st-CYS 23	
BC-LOOP	27–38	12 (or less)		CDR1-IMGT
C-STRAND	39–46	8	CONSERVED-TRP 41	FR2-IMGT
C’-STRAND	47–55	9		
C’C”-LOOP	56–65	10 (or less)		CDR2-IMGT
C”-STRAND	66–74	9 (or 8 if gap at 73)		FR3-IMGT
D-STRAND	75–84	10 (or 8 if gaps at 81, 82)		
E-STRAND	85–96	12	hydrophobic 89	
F-STRAND	97–104	8	2nd-CYS 104	
FG-LOOP	105–117	13 (or less, or more)		CDR3-IMGT
G-STRAND	118–128	11 (or 10)	V-DOMAIN J-PHE 118 or J-TRP 118 ^d^	FR4-IMGT

^a^ IMGT^®^ labels (concepts of description) are written in capital letters. ^b^ in number of amino acids (or codons). ^c^ Residue@Position is a IMGT^®^ concept of numerotation that numbers the position of a given residue (or that of a conserved property amino acid class), based on the IMGT unique numbering. ^d^ In the IG and TR V-DOMAIN, the G-STRAND (or FR4-IMGT) is the C-terminal part of the J-REGION, with J-PHE or J-TRP 118 and the canonical motif F/W-G-X-G (J-MOTIF) at positions 118–121.

**Table 2:** C domain strands, turns and loops, IMGT positions and lengths, based on the IMGT unique numbering for C domain (C-DOMAIN and C-LIKE-DOMAIN) [7].

C Domain Strands, Turns and Loops ^a^	IMGT Positions	Lengths ^b^	Characteristic Residue@Position ^c^
A-STRAND	1–15	15 (14 if gap at 10)	
AB-TURN	15.1–15.3	0–3	
B-STRAND	16–26	11	1st-CYS 23
BC-LOOP	27–31 34–38	10 (or less)	
C-STRAND	39–45	7	CONSERVED-TRP 41
CD-STRAND	45.1–45.9	0–9	
D-STRAND	77–84	8 (or 7 if gap at 82)	
DE-TURN	84.1–84.7 85.1–85.7	0–14	
E-STRAND	85–96	12	hydrophobic 89
EF-TURN	96.1–96.2	0–2	
F-STRAND	97–104	8	2nd-CYS 104
FG-LOOP	105–117	13 (or less, or more)	
G-STRAND	118–128	11 (or less)	

^a^ IMGT^®^ labels (concepts of description) are written in capital letters. ^b^ in number of amino acids (or codons). ^c^ Residue@Position is a IMGT^®^ concept of numerotation that numbers the position of a given residue (or that of a conserved property amino acid class), based on the IMGT unique numbering.

**Table 3:** Correspondence between the IGHG1 alleles and G1m alleles.

IGHG1 Alleles	G1m Alleles *^a^*		IMGT Amino Acid Positions *^b^*								Populations [18]
	Allotypes	Isoallotypes *^c^*	CH1		CH3						
			103	120	12	14	101	110	115	116	
				G1m17/nG1m17	G1m1/nG1m1		/G1m27	/G1m2	/G1m28-		
			G1m3 *^d^*								
IGHG1*01 *^e^*, IGHG1*02 *^e^*, IGHG1*05 *^e^*	G1m17,1		I	K	D	L	V	A	H	Y	Caucasoid Negroid Mongoloid
IGHG1*03	G1m3	*nG1m1,* *nG1m17*	I	R	E	M	V	A	H	Y	Caucasoid
IGHG1*04	G1m17,1,27		I	K	D	L	I	A	H	Y	Negroid
IGHG1*05p *^f^*	G1m17,1,28		I	K	D	L	V	A	R	Y	Negroid
IGHG1*06p *^f^*	G1m,17,1,27,28		I	K	D	L	I	A	R	Y	Negroid
IGHG1*07p *^f^*	G1m17,1,2		I	K	D	L	V	G	H	Y	Caucasoid Mongoloid
IGHG1*08p *^f^*	G1m3,1	*nG1m17*	I	R	D	L	V	A	H	Y	Mongoloid

*^a^* In Negroid populations, the G1m17,1 allele frequently includes G1m27 and/or G1m28, leading to three additional G1m alleles, G1m17,1,27, G1m17,1,28 and G1m17,1,27,28 [18]. *^b^* Amino acids corresponding to G1m allotypes are shown in bold. *^c^* The nG1m1 and nG1m17 isoallotypes present on the Gm1-negative and Gm-17 negative gamma-1 chains (and on other gamma chains) are shown in italics. *^d^* The presence of R120 is detected by anti-nG1m17 antibodies whereas the simultaneous presence of I103 and R120 in the gamma1 chains is detected by anti-Gm3 antibodies [18]. *^e^* The IGHG1*01, IGHG1*02 and IGHG1*05 alleles only differ at the nucleotide level (codon 85.1 in CH2 of *02 and *05 differs from *01, codon 19 in CH1 and codon 117 in CH3 of *05 differ from *01 and *02). *^f^* IGHG1*05p, IGHG1*06p, IGHG1*07p and IGHG1*08p amino acids are expected [18] but not yet sequenced at the nucleotide level and therefore these alleles are not shown in IMGT Repertoire, Alignments of alleles: *Homo sapiens* IGHG1 (http://www.imgt.org).

**Table 4:** IMGT engineered variant nomenclature. Examples of IGHG1 variants involved in antibody-dependent cellular (ADCC), antibody-dependent cellular phagocytosis (ADCP), complement-dependent cytotoxicity (CDC), half-life increase, half-IG exchange, B cell inhibition, and knobs-into-holes are shown. Amino acid positions, correspondence with the EU numbering and bibliographical references are quoted at http://www.imgt.org/IMGTeducation/Tutorials/IGandBcells/_UK/IGproperties/Tableau3.html.

IMGT Engineered Variant Nomenclature	IGHG Gene Variant Description				Property Modifications		
	CH2	AA and IMGT Position in CH2 of IGHG Gene Variant	CH3	AA and IMGT Position in CH3 of IGHG Gene Variant	ADCC Enhancement or Reduction, ADCP Enhancement, B Cell Inhibition	CDC Enhancement or Reduction	Half-IG Exchange Reduction, Half-Life Increase, Knobs-Into-Holes
Homsap IGHG1v1	CH2	P1.4			ADCC reduction		
Homsap IGHG1v2	CH2	V1.3			ADCC reduction		
Homsap IGHG1v3	CH2	A1.2			ADCC reduction		
Homsap IGHG1v4	CH2	A114			ADCC reduction	CDC reduction	
Homsap IGHG1v5	CH2	W109			ADCC reduction	CDC enhancement	
Homsap IGHG1v6	CH2	A85.4, A118, A119			ADCC enhancement		
Homsap IGHG1v7	CH2	D3, E117			ADCC enhancement		
Homsap IGHG1v8	CH2	D3, L115, E117			ADCC enhancement	CDC reduction	
Homsap IGHG1v9	CH2	L7, P83, L85.2, I88	CH3	L83	ADCC enhancement		
Homsap IGHG1v10	CH2	Y1.3, Q1.2, W1.1, M3, D30, E34, A85.4			ADCC enhancement		
Homsap IGHG1v11	CH2	E34, D109, M115, E119			ADCC enhancement		
Homsap IGHG1v12	CH2	A1.1, D3, L115, E117			ADCC enhancement		
Homsap IGHG1v13	CH2	A1.1, D3, E117			ADCP enhancement		
Homsap IGHG1v14	CH2	A1.3, A1.2			ADCC reduction	CDC reduction	
Homsap IGHG1v15	CH2	S118				CDC enhancement	
Homsap IGHG1v16	CH2	W109, S118				CDC enhancement	
Homsap IGHG1v17	CH2	E29, F30, T107				CDC enhancement	
Homsap IGHG1v18			CH3	R1, G109, Y120		CDC enhancement	
Homsap IGHG1v19	CH2	A34				CDC reduction	
Homsap IGHG1v20	CH2	A105				CDC reduction	
Homsap IGHG1v21	CH2	Y15.1, T16, E18					Half-life increase
Homsap IGHG1v22	CH2	Y15.1, T16, E18	CH3	K113, F114, H116			Half-life increase
Homsap IGHG1v23	CH2	E1.2			ADCC reduction	CDC reduction	
Homsap IGHG1v24			CH3	L107, S114			Half-life increase
Homsap IGHG1v25	CH2	E29, F113			B cell inhibition		
Homsap IGHG1v26			CH3	Y22, T86			Knobs-into-holes

## Availability and Citation

Authors who use IMGT^®^ databases and tools are encouraged to cite this article and to quote the IMGT^®^ Home page, http://www.imgt.org. Online access to IMGT^®^ databases and tools are freely available for academics and under licences and contracts for companies.
